# Alzheimer's Disease and Cognitive Frailty: Novel Therapeutic Technologies

**DOI:** 10.1155/2016/7242651

**Published:** 2016-07-25

**Authors:** Grazia D'Onofrio, Zhuowei Yu, Orestes V. Forlenza

**Affiliations:** ^1^Geriatric Unit and Laboratory of Gerontology and Geriatrics, Department of Medical Sciences, IRCCS “Casa Sollievo della Sofferenza”, San Giovanni Rotondo, 71013 Foggia, Italy; ^2^Shanghai Institute of Geriatrics and Gerontology, Shanghai Key Laboratory of Clinical Geriatrics, Department of Geriatrics, Huadong Hospital, and Research Center of Aging and Medicine, Shanghai Medical College, Fudan University, Shanghai 200040, China; ^3^Laboratory of Neuroscience (LIM-27), Department and Institute of Psychiatry, Faculty of Medicine, University of São Paulo, 05403-010 São Paulo, SP, Brazil

Worldwide, 46.8 million people have dementia, and every year there are over 9.9 million new diagnosed cases [1], with an increase of the economic impact and cost of the 35.4% from 2010 [1]. Alzheimer's disease (AD) is the most common form of dementia [2] and represents one of the major causes of disability, dependency, burden, and stress of caregivers increasing institutionalization among older people worldwide [3].

A precursor of neurodegenerative processes may be represented by Cognitive Frailty (CF) that includes reversible and potentially reversible subtypes [4].

Risk factors for CF and AD appear to change with age. Most very elderly individuals have beta-amyloid (A*β*) plaques within their brains, indicating that AD pathology may be present in asymptomatic elders and that these individuals will ultimately develop clinical symptoms of AD if they live long enough. According to the preclinical AD criteria, therefore, it is difficult to see how brain amyloidosis and brain aging could be considered separate entities arising through independent mechanisms.

In this special issue, investigators reported studies into the subject from all over the world (i.e., Switzerland, Germany, Egypt, China, Spain, Australia, Italy, Brazil, and USA).

They contributed to increasing the knowledge on to individualize genetic and clinic mechanisms of AD and CF considering age-related and multidimensional approaches to the purpose of appropriate and personalized treatment.

In a preclinical model, S. AbdAlla et al. subjected aged rats to chronic unpredictable mild stress, which is known to enhance the development of AD-related neuropathological features, and showed that four weeks of chronic mild stress induced a strong upregulation of the hippocampal angiotensin-converting enzyme (ACE), both at gene expression and at protein levels. The authors reported that ACE inhibition targets neurodegeneration triggered by environmental stress.

C. Ma et al. started from the assumption that advancing age, chronic inflammation, oxidative damage, and disorders of lipid metabolism are positively linked to the late-life cognitive impairment. This study demonstrated that age, levels of fundus atherosclerosis, serum biomarkers peroxidase, interleukin-6, serum levels of high-density lipoprotein cholesterol, ApoA2, and ApoC2 were significantly related to cognitive status. Moreover, ApoA1 and ApoA2 were found to be possible risk factors of cognitive impairment and late-life dementia.

J. A. Monge-Argilés et al. analyzed cerebrospinal fluid (CSF) biomarkers and tau/A*β* ratios in MCI patients and control subjects, using ELISA methodology. This study contributed to evaluation of the association between apolipoprotein E (ApoE) genotype and CSF levels of AD biomarkers and the influence of ApoE genotype on the development of AD in a Spanish population.

G. Lyons et al. described the “Deep Assessment,” which is a novel multifaceted framework for delivering a more comprehensive and authentic assessment of the internal states of people with severe cognitive impairments who are unable to self-report. This paper suggested how Deep Assessment can be applied to people with advanced AD to develop others' understanding of their inner states and to help improve their quality of life. Moreover G. Lyons et al. discussed the potential utility and efficacy of this technique for this population and also proposed other human conditions that may benefit from research using a Deep Assessment approach.

F. Panza et al. reviewed tau-centric targets and drugs in clinical development for the treatment of AD and reported that methylene blue seems to be an inhibitor of the tau protein aggregation.

Finally, A. M. de Oliveira et al. reviewed studies of nonpharmacological interventions published in the last 10 years and reported that such approaches may help reduce behavioral and psychological symptoms of dementia (BPSD) such as agitation, psychotic symptoms, and apathy. This study highlighted the role of nonpharmacological interventions programs in the clinical management of BPSD, as an alternative to (or in combination with) conventional pharmacological treatments (e.g., antipsychotics and benzodiazepines) that may elicit undesired side effects.

The pharmacologic treatments, even if they are supported by several studies, deliver limited symptomatic benefits, so the provision of nonpharmacological treatments in addition to standard outpatient care is an asset of good clinical practice.

Therefore prognostic evaluation of AD patients plays a key role in the decision analyses of care processes including the organization of social health care system, the support to families, caregivers, and patients as well as the choice of appropriate treatment.

*Grazia D'Onofrio* *Zhuowei Yu* *Orestes V. Forlenza*
